# One-dose intradermal rabies booster enhances rabies antibody production and avidity maturation

**DOI:** 10.1007/s00430-024-00791-2

**Published:** 2024-05-18

**Authors:** Chidchamai Kewcharoenwong, Saranta Freeouf, Arnone Nithichanon, Wilaiwan Petsophonsakul, Sakorn Pornprasert, Woottichai Khamduang, Tadaki Suzuki, Taishi Onodera, Yoshimasa Takahashi, Ganjana Lertmemongkolchai

**Affiliations:** 1 Department of Medical Technology, Faculty of Associated Medical Sciences, Chiang Mai University, Chiang Mai, Thailand; 2 The Centre for Research & Development of Medical Diagnostic Laboratories, Faculty of Associated Medical Sciences, Khon Kaen University, Khon Kaen, Thailand; 3 Lanna Dog Welfare, Chiang Mai, Thailand; 4 Department of Microbiology, Faculty of Medicine, Khon Kaen University, Khon Kaen, Thailand; 5 Department of Pathology, National Institute of Infectious Diseases, Tokyo, Japan; 6 Research Center for Drug and Vaccine Development, National Institute of Infectious Diseases, Tokyo, Japan

**Keywords:** Rabies vaccination, Rabies antibody, Avidity maturation, One-dose ID booster

## Abstract

The incidence of rabies in Thailand reached its peak in 2018 with 18 human deaths. Preexposure prophylaxis (PrEP) vaccination is thus recommended for high-risk populations. WHO has recently recommended that patients who are exposed to a suspected rabid animal and have already been immunized against rabies should receive a 1-site intradermal (ID) injection of 0.1 mL on days 0 and 3 as postexposure prophylaxis (PEP). In Thailand, village health and livestock volunteers tasked with annual dog vaccination typically receive only a single lifetime PrEP dose and subsequent boosters solely upon confirmed animal bites. However, the adequacy of a single PrEP dose for priming and maintaining immunity in this high-risk group has not been evaluated. Therefore, our study was designed to address two key questions: (1) sufficiency of single-dose PrEP—to determine whether a single ID PrEP dose provides adequate long-term immune protection for high-risk individuals exposed to numerous dogs during their vaccination duties. (2) Booster efficacy for immune maturation—to investigate whether one or two additional ID booster doses effectively stimulate a mature and sustained antibody response in this population. The level and persistence of the rabies antibody were determined by comparing the immunogenicity and booster efficacy among the vaccination groups. Our study demonstrated that rabies antibodies persisted for more than 180 days after cost-effective ID PrEP or the 1st or the 2nd single ID booster dose, and adequate antibody levels were detected in more than 95% of participants by CEE-cELISA and 100% by indirect ELISA. Moreover, the avidity maturation of rabies-specific antibodies occurred after the 1st single ID booster dose. This smaller ID booster regimen was sufficient for producing a sufficient immune response and enhancing the maturation of anti-rabies antibodies. This safe and effective PrEP regimen and a single visit involving a one-dose ID booster are recommended, and at least one one-dose ID booster regimen could be equitably implemented in at-risk people in Thailand and other developing countries. However, an adequate antibody level should be monitored before the booster is administered.

**Supplementary Information:**

The online version contains supplementary material available at 10.1007/s00430-024-00791-2.

## Background

Rabies is a fatal but preventable viral infection. This deadly disease causes approximately 59,000 deaths worldwide annually, and 59% of all such deaths occur on the Asian continent [1]. The incidence of rabies in Thailand reached its peak in 2018 with 18 human deaths [2]. In addition to Bangkok, Chiang Mai Province is one of the most important areas in Thailand; thus, local governments have adopted an intensive policy to eliminate human and rabies deaths and to control rabies-positive animals. However, a case in which canine rabies reemerged in Chiang Mai was recently reported in February 2023 [3].

Postprophylaxis (PEP) consists of a set of rabies vaccines (RVs) together with rabies immunoglobulin (RIG) injections and is crucial for nonimmunized humans after any bite or injury from dogs, cats or other mammals. In particular, RIG should be administered for severe category III exposures [4]. However, preexposure prophylaxis (PrEP) vaccination is also recommended for high-risk individuals who have not been vaccinated by PEP or PrEP previously according to the WHO recommendation. High-risk groups for rabies exposure include animal healthcare personnel, wildlife officers, veterinarians, veterinary students, hospital healthcare workers, and certain laboratory workers, particularly in rabies-endemic areas. These individuals face a considerable risk of encountering infected animals, especially in regions where rabies is prevalent [5]. RV administration varies in terms of the time schedule and route of injection (intramuscular or intradermal). Intradermal injection has been reported to give an immune boost equivalent to that of intramuscular injection but requires a lower dose for both PrEP and PEP vaccination. Therefore, the intradermal method is more appropriate for areas or regions with an insufficient supply of the vaccine and in which the vaccine can be distributed to more at-risk exposure groups.

However, to obtain the full benefits of intradermal vaccination, nurses must be well trained to be able to administer the full intradermal instillation of the vaccine rather than accidentally injecting the vaccine into the subcutaneous layer. Nonetheless, both routes of injection can stimulate rapid immune responses after booster vaccination to maintain the level of antibody against the rabies virus [6–8]. The current guideline policy for rabies PrEP vaccination for cost-effectiveness according to the Thai Red Cross 2018 corresponds to the 2017 WHO guidelines. For nonvaccinated personnel, one dose of 0.5 mL (2.5 IU) intramuscularly (IM) was given at each day 0 and 7 (for a total of 1 mL) and one intradermal (ID) injection, involving two doses of 0.1 mL (2–2), was given on each arm on days 0 and 7 (for a total of 0.4 mL). Recently, the Thai government issued a letter to local governments stating that high-risk groups such as villages or livestock volunteers should only receive the 2-dose 2–2 WHO regimen once, without a booster, except in the case of a dog bite. Furthermore, if any primary rabies vaccinators are exposed to a suspicious rabid animal, a complete booster regimen is suggested [9]. The purpose of administering PrEP via the 2-dose ID (2–2) schedule is to reduce the cost of using rabies immunoglobulin (Ig) and complete vaccination (4 or 5 doses ID or IM) in the event of a dog bite for people who have never had PrEP before. For individuals who have previously received PrEP, the Thai Red Cross and WHO guidelines specify that individuals with prior PrEP exposure should receive postexposure prophylaxis (PEP) differently based on the timing of their last PrEP dose. If the previous PrEP dose was less than 6 months prior, patients should receive one dose of intramuscular (IM) or 0.1 mL of intradermal (ID) vaccine. If the previous PrEP dose was more than 6 months prior, patients should receive two doses of either the IM or ID vaccine. However, if the person has no previous vaccination, complete treatment with rabies Ig and complete vaccination (4 or 5 doses ID or IM) are needed. Therefore, PrEP is recommended for high-risk groups or people living in endemic areas with a high incidence of rabies.

To improve compliance with high efficacy, progressive booster regimens requiring fewer injections have been developed. Fewer injection regimens would also facilitate rabies vaccination among high-risk village volunteers who are at risk of being exposed to rabies virus every year. As such, one ID dose of the booster regimen was applied to two high-risk village volunteers who had been vaccinated with cost-saving PrEP one year before vaccination and who showed an adequate level of anti-rabies antibody on day 30 after vaccination [10]. However, the boosting ability and persistence of adequate antibodies in response to a single ID dose of booster agent are still underdetermined. Booster regimens are recommended by the WHO as an extra precaution only for people whose occupation puts them at continual or frequent risk of exposure. A booster shot may be necessary for village health volunteers to maintain neutralizing antibody levels for at least a year, as they are exposed to dogs when they go for vaccination.

On the other hand, some high-risk groups in Thailand, especially veterinarians and veterinary students, routinely receive PrEP and annual boosters with or without exposure. Thus, determining the presence of anti-rabies antibodies is essential for ensuring that patients have adequate levels of antibiotics to protect themselves. Moreover, if they already have adequate levels, booster injection is unnecessary. Even if the WHO recommends that only those at risk whose level is below an arbitrary level should receive a booster, the gold standard detection of anti-rabies antibody, rapid fluorescent focus inhibition test (RFFIT), is difficult to access in Thailand and most Asian countries. The cost effective easy competitive ELISA (CEE-cELISA) could be an alternative assay for evaluating mass rabies vaccination rapidly at a low cost as well as for detecting anti-rabies antibodies [11].

In this study, we provided a cost-effective PrEP ID rabies vaccination for high-risk village volunteers followed by one ID-dose rabies booster annually for 2 years. Here, we focus on the efficacy of a single-dose rabies booster in terms of antibody adequateness and antibody avidity as well as adverse effects after individuals receive cost-saving primary and booster vaccination. Moreover, our study was designed to address two crucial questions regarding PrEP and booster regimens for high-risk individuals, such as village health volunteers, who encounter various dogs: (1) sufficiency of single-dose PrEP followed by a single-visit, one-site ID booster injection—this simplified schedule could significantly improve cost-effectiveness and program feasibility for these individuals. (2) Antibody response to a single booster—we hypothesized that one or two boosters might be necessary for optimal antibody maturation, ensuring sufficient protection against potential rabies exposure.

By carefully evaluating the effectiveness of a single-dose PrEP and single-visit ID booster, we seek to contribute to the development of optimized rabies prevention strategies for high-risk individuals in resource-limited settings, balancing cost-effectiveness with robust protection.

## Results

### Study design, participant demographics and safety

**Fig. 1:**
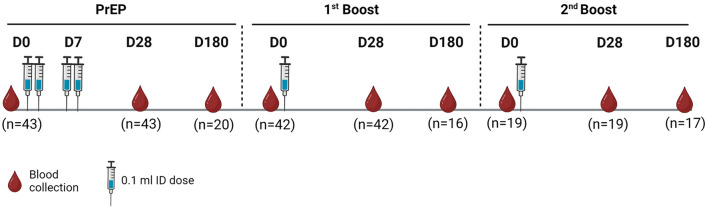
Schematic diagram of intradermal rabies vaccination and blood collection. *D* day, *ID* intradermal, *PrEP* preexposure prophylaxis

All participants received at least two ID doses of purified Vero cell rabies vaccine (PVRV, > 2.5 IU/IM dose; 0.5 mL/ampoule; Speeda^®^, China) (2 × 0.1 mL) on D0 and D7 for the PrEP regimen (for a total of 0.4 mL) and were categorized into three groups: the participants in the PrEP group received only the PrEP regimen (n = 43). Participants in the 1st booster group received the first ID booster dose the year after PrEP (n = 42), and participants in the 2nd booster group received the second ID booster dose the year after the 1st ID booster dose (n = 19) (Fig. 1). Most of the participants in the three groups were males (58.1, 81.0 and 100.0%), and the median ages at enrolment were 52.0 (IQR 45.0–60.0), 57.5 (IQR 51.2–63.0) and 61.0 (IQR 56.5–65.0) years for the PrEP, the 1st booster and the 2nd booster group, respectively. The most common underlying diseases in all groups were diabetes mellitus, hypertension and hyperlipidaemia (Table 1).

**Table 1:** Characteristics of the participants

Participant’s characteristics	PrEP (n = 43)	1st booster (n = 42)	2nd booster (n = 19)
Sex, n (%)			
Male	25 (58.14)	34 (80.95)	19 (100.00)
Female	18 (41.86)	8 (19.05)	0 (0.00)
Median age, in years (IQR)	52 (45.00–60.00)	57.5 (51.25–63.00)	61 (56.50–65.00)
Underlying diseases, n (%)			
Diabetes mellitus	4 (9.30)	7 (16.67)	6 (31.58)
Hypertension	6 (13.95)	10 (23.81)	6 (31.58)
Hyperlipidaemia	7 (16.28)	7 (16.67)	4 (21.05)
Other diseases			
Cardiovascular disease	1 (2.33)	1 (2.38)	1 (5.26)
Gout	2 (4.65)	1 (2.38)	–
Thyroid	2 (4.65)	–	–
Migraine	1 (2.33)	1 (2.38)	–
Emphysema	1 (2.33)	–	–

Overall, among participants who reported adverse events following immunization (AEFIs) in the 2nd booster group, 2 (10.5%) reported more than one symptom, a significant decrease when compared with 19 (45.2%) in the PrEP group and 16 (39.0%) in the 1st booster group (Table 2). AEFIs, especially systemic reactions, fever and fatigue, tended to decrease after the 1st and 2nd booster doses. The most common symptoms in the PrEP group were muscle pain (14.0%), fever (11.6%) and itching (9.3%), while those in the 1st booster group were local AEFIs associated with injection site itching (16.7%), swelling (11.9%) and muscle pain (9.5%). However, no severe AEFI was observed in this study.

**Table 2:** Percentage of participants who reported adverse events at D28 after vaccination

Adverse event n (%)	PrEP (n = 43)	1st boost (n = 42)	2nd boost (n = 19)
Fever	5 (11.63)	1 (2.38)	–
Fatigue	2 (4.65)	–	–
Muscle pain	6 (13.95)	4 (9.52)	–
Bruise	1 (2.33)	–	–
Rash	1 (2.33)	2 (4.76)	1 (5.26)
Itching	2 (4.65)	7 (16.67)	–
Swelling	2 (4.65)	5 (11.90)	1 (5.26)

### Immunogenicity and booster effectiveness

**Fig. 2:**
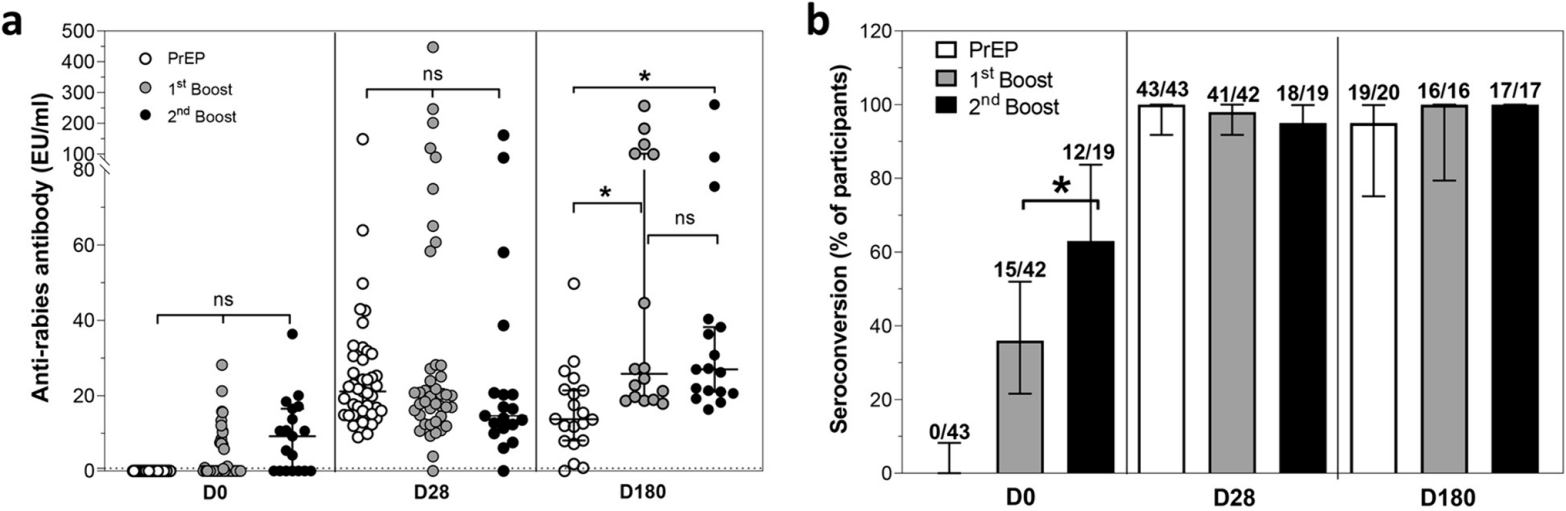
Immunogenicity and booster effectiveness. **a** Rabies neutralizing antibody levels at each time point were measured by CEE-cELISA; each data point represents the median with 95% CI for each individual; the horizontal dashed line indicates 0.7 EU/mL (indicator of adequate vaccination); statistical analysis was performed using Dunn’s multiple comparisons test; **b** seroconversion rates at each time point; each bar represents the percentage with 95% CI of participants who had an antibody level ≥ 0.7 EU/mL in each group; the white, grey and black dots or bars indicate the PrEP, 1st booster and 2nd booster groups, respectively; statistical analysis was performed using a Chi-square test; *p < 0.05; no asterisk and *ns* nonsignificant

The anti-rabies antibody levels in the three groups, PrEP and the 1st and 2nd booster groups, on D0, D28 and D180 were detected by CEE-cELISA. The lowest concentration of an anti-rabies antibody that could be detected by the CEE-cELISA was approximately 0.7 EU/mL, which was comparable with the REFFT result at 0.5 IU/mL [11]. On D0 before immunization and D28 after immunization, the median rabies antibody level was not different among the three groups; however, on D180, the median level in PrEP (16.5 EU/mL [95% CI 7.2–24.7 EU/mL) was significantly lower than that in the 1st booster group (25.8 EU/mL [95% CI 18.9–101.2 EU/mL]) and the 2nd booster group (27.0 EU/mL [95% CI 26.0–38.2 EU/mL]) (Fig. 2a).

The persistence of rabies antibodies was first observed on D0. The measurable antibodies on D0 in the 1st and 2nd booster groups persisted approximately one year after PrEP and the 1st booster vaccination. Among the participants who had an adequate antibody level ≥ 0.7 EU/mL on D0 in the 2nd booster group, 63.0% [95% CI 38.3–83.7%] had an adequate antibody level significantly greater than that in the 1st booster group (36.0% [95% CI 21.6–52.0%]). The percentage of participants who had an inadequate antibody level (< 0.7 EU/mL) after PrEP vaccination for one year (D0 of the 1st booster group) was 64.0% [95% CI 48.0–78.4], while that at D28 was 2.0% [95% CI 0.1–12.3%] after the 1st boost. Similarly, the percentage of patients who underwent a 1st boost for one year (D0 of the 2nd booster group) was 37.0% [95% CI 16.3–61.6%] when the percentage at D28 was 5% [95% CI 0.1–26.0%]. These results imply that even if the level of antibody after PrEP or the 1st boost vaccination declines to an inadequate level, the antibody level can be restored after boosting. All groups exhibited a high seroconversion rate (≥ 95%) on D28 and D180 after participants received PrEP or booster doses (Fig. 2b); however, the antibody level in the PrEP group decreased significantly on D180 (Fig. 2a).

To estimate the half-life of the antibodies in response to rabies vaccination, response profiles that showed a decrease in antibody levels over at least two consecutive sampling points in the PreP and 1st booster groups were selected, and one-phase exponential decay curves fitted to the decay phase of the selected response profiles were generated (Additional Fig. 1). The mean half-life of the antibodies in the PrEP group was 118.0 days, whereas that in the 1st booster group was 121.4 days. The half-lives of the responses did not differ significantly between the two groups.

Taken together, the data indicated that at least one ID booster dose was sufficient to increase the adequate antibody concentration after a participant had been vaccinated with the PrEP regimen for one year. Moreover, one ID booster dose was used when measuring the persistence of an adequate rabies antibody level for more than 180 days and to increase the percentage of seroconversion on D0 in the next year.

### Pattern of antibody response after the 1st and 2nd booster doses

Among the participants in the 1st and 2nd booster groups, 19 were the same individuals who were enrolled in different years, and the pattern of antibody response to both recombinant rabies virus glycoprotein (rRVGP) and the rabies vaccine was observed by CEE-cELISA and indirect ELISA. The RVGP plays important roles in the early and late phases of virus replication [12]. The patterns of antibody response on D0 and D28 after the 1st and 2nd booster doses were similar and correlated between the CEE-cELISA and indirect ELISA methods (Additional Fig. 2a–c and Additional Fig. 3).

Only one male participant was negative for an antibody response on D28 after the 1st and 2nd booster doses according to CEE-cELISA, but antibodies against both the rRVGP and the rabies vaccine were detectable via indirect ELISA. However, the detectable antibody level of this participant was low on D28 after the 1st booster dose (46.9 AU/mL; median across this group, 114.1 AU/mL [% CI 77.1–148 AU/mL]), and after the 2nd booster dose, a level of 38.6 AU/mL was detected (median across this group, 117.8 AU/mL [% IC: 75.7–170.8 AU/mL]) (Additional Fig. 2a–c). Samples taken on D28 following receipt of the 2nd booster dose confirmed the neutralizing activity of the rabies antibody by the Rapid Fluorescent Foci Inhibition Test (RFFIT) performed at the National Institute of Infectious Diseases, Tokyo, Japan, and the RFFIT data were > 0.5 IU/mL (indicator of adequate vaccination). Thus, this participant was a low responder to the rabies vaccine and was able to produce an adequate antibody response after immunization with one ID booster dose.

### Anti-rabies antibody and avidity indices in response to recombinant rabies virus glycoprotein and the rabies vaccine on D28 after vaccination

**Fig. 3:**
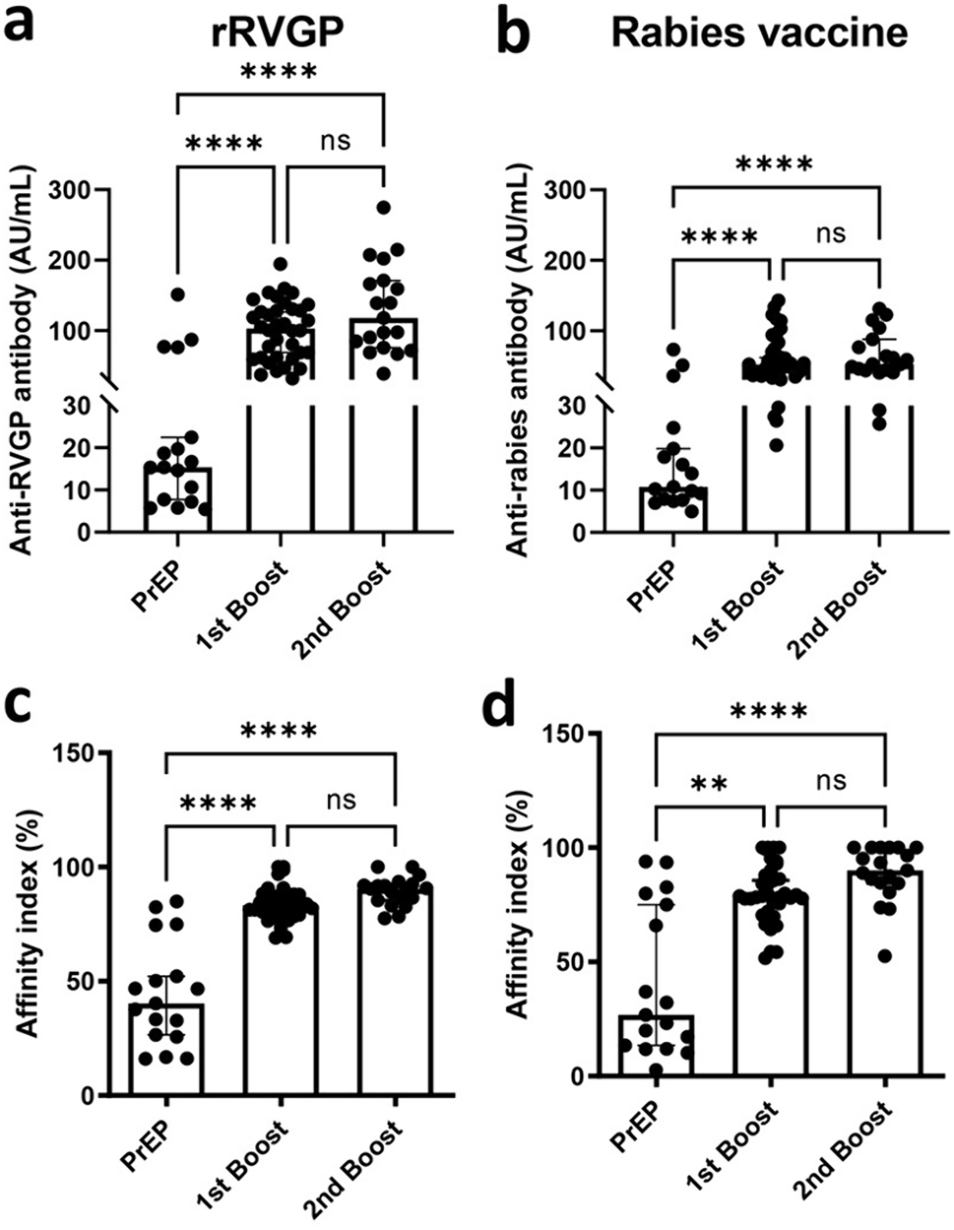
Anti-rabies IgG antibody and avidity index in response to recombinant rabies virus glycoprotein and the rabies vaccine at D28. IgG antibody levels in the **a** recombinant rabies virus glycoprotein (rRVGP) and **b** rabies vaccine groups measured by indirect ELISA; percentage of avidity indices in response to the **c** rRVGP and **d** rabies vaccines; the samples were selected from the PrEP (n = 17), 1st booster (n = 36) and 2nd booster (n = 19) groups; the bars indicate the medians with 95% CIs, and each dot represents the value of each sample; statistical analysis was performed using Dunn’s multiple comparisons test; ***p < 0.001; **p < 0.01; *ns* nonsignificant

To determine the strength of the binding interaction between the antigen and the antibody, the avidity index of the anti-rabies antibody was measured. Anti-rabies antibody was observed by indirect ELISA with or without urea treatment. Anti-rabies IgG antibody levels in response to the rRVGP and rabies vaccine on D28 in the three groups were measured via indirect ELISA. The median IgG antibody concentration against the rRVGP significantly increased in the 1st booster (102.8 AU/mL [95% CI 68.8–126.2 AU/mL]) and 2nd booster (117.8 AU/mL [95% CI 75.7–170.8 AU/mL]) groups compared with the PrEP group (15.4 AU/mL [95% CI 7.7–22.5 AU/mL]) (Fig. 3a). Similarly, the median level of IgG antibody to the rabies vaccine was significantly elevated in the 1st booster (51.6 AU/mL [95% CI 40.2–61.8 AU/mL]) and 2nd booster (52.6 AU/mL [95% CI 43.8–87.6 AU/mL]) groups compared with the PrEP group (10.8 AU/mL [95% CI 8.0–19.8 AU/mL]) (Fig. 3b).

As shown for the antibody level, the avidity index (expressed in %) increased significantly in the 1st booster group (83.4% [95% CI 81.1–87.4%) and the 2nd booster group (90.5% [95% CI 85.4–92.6%) compared with the PrEP group (40.3% [95% CI 26.5–52.2%) in response to the rRVGP (Fig. 3c). Similarly, for the rabies vaccine, the avidity index was significantly greater in the 1st booster group (78.8% [95% CI 77.7–85.7%) and the 2nd booster group (90.1% [95% CI 84.5–100.0%) than in the PrEP group (26.8% [95% CI 13.7–75.1%) (Fig. 3d). These data indicated that the elevated level and avidity maturation of rabies-specific antibodies occurred during vaccination, especially after the 1st booster.

## Discussion

Our study provides valuable evidence of the efficacy of cost-saving 1-week ID vaccination (two doses on D0 and D7) as a booster regimen as well as the efficacy of PrEP and a single-dose ID, which could be used in high-risk individuals or other settings when a booster dose is needed. Our data showed that one-dose ID rabies was highly effective at boosting the immune response, resulting in the development of antibody levels and avidity maturation after the 1st booster dose in all participants, including the participant who was a low responder. Moreover, our study provides important evidence about the long-term persistence of antibodies after the 2nd single ID booster dose from increasing the percentage of seroconversion for at least one year. The benefits of these PrEP regimens and a one-dose ID booster regimen include the ability to provide PrEP and a booster dose to high-risk individuals; additional benefits include the use of VHVs and LVs, convenience, cost savings and dose savings (during times of vaccine shortage).

Overall, there were no safety concerns in any of these study groups. PrEP and simulated injections via the ID route were properly tolerated and had satisfactory safety profiles. The adverse events after each injection tended to decrease after the 1st and 2nd booster doses. The safety and tolerability of the treatments in the study groups appeared to be consistent with those of previous studies using these vaccines and regimens [10, 13].

This study was performed to investigate the humoral immune response following primary and booster vaccination with the rabies virus glycoprotein and rabies vaccine in high-risk village volunteers. We confirmed that the participants were primed after receiving a cost-saving rabies PrEP vaccination independent of the achievement of seroconversion (anti-rabies antibody ≥ 0.7 EU/mL) as determined using the CEE-cELISA procedures from our previous study [11]. Our other study provided evidence that CEE-cELISA is suitable for monitoring antibodies against the rabies virus in the serum of humans compared with RFFIT results and revealed a very strong correlation between CEE-cELISA and RFFIT data [10]. Moreover, RVGP serology ELISA has been proven to be able to measure neutralizing antibodies in the sera of vaccinated humans in a double-blind test by the RFFIT, and the specificity and sensitivity were 100% and 91.1%, respectively [14]. Additional Fig. 3 shows the correlation between CEE-cELISA and indirect ELISA results, with a p value < 0.0001, in response to both the rRVGP and the rabies vaccine. These data imply that CEE-cELISA and indirect ELISA, especially for the rRVGP, are reliable for measuring the antibody response following vaccination.

Moreover, a single ID booster dose was sufficient to increase the level of anti-rabies antibody. These data are consistent with those of a previous study of Thai VHVs and LVs [10]. Another study showed that rabies antibodies persisted for many years after ID PrEP, and an adequate antibody response could be expected 7 days after a single ID booster dose in more than 99% of participants; however, the participants in this study were vaccinated with a standard schedule (three 0.1-ml doses) and a modified schedule (five 0.1-mL doses) [15].

We followed the antibody response on D180 from the participants who received PrEP, the 1st booster and the 2nd booster dose and found that antibody levels in the PrEP group were significantly lower than those in the participants who received the 1st and 2nd booster doses. However, the percentage of seroconversion in participants after they received the 1st booster dose for one year was significantly greater than that in participants who received only PrEP as a prime-dose vaccination for one year. Similarly, a rapid decrease in antibody levels 1 year after immunization and no further increase in antibody levels following a third booster immunization have been reported after intramuscular dose vaccination [16]. Thus, our antibody level data indicated that cost-saving rabies PrEP and a single ID booster dose were sufficient to prime and restimulate the immune response. In a previous study, the participants were followed for 10 years after receiving either 2 or 3 doses of PrEP regimens and a booster dose one year later. The results showed that more than 95% of all participants who received the PrEP regimen followed by one dose of vaccine one year later still had adequate anti-rabies antibody levels 10 years after receiving their initial series [17]. Consistently, our study provides evidence for an increased avidity index after the 1st booster dose. Yearly boosters may be beneficial for unrecognized risk in high-risk people in Thailand; however, from our results, at least one booster one year later is recommended.

Moreover, we determined the avidity indices in response to the rRVGP and rabies vaccines to investigate antibody maturation in the PrEP, 1st booster and 2nd booster groups. Our data showed that the avidity index was significantly greater after the 1st one-dose ID booster, while no difference was observed after the 2nd one-dose ID booster compared with the 1st booster. These results implied that only one single ID booster was sufficient to activate the maturation of anti-rabies antibodies, especially the anti-rRVGP antibody, which interacts with the RVGP epitope in the viral protein for neutralization [18]. To our knowledge, this is the first study showing avidity maturation of antibodies elicited by one-dose ID rabies vaccination.

However, we observed a difference in antibody levels between the PrEP and booster groups when antibodies were detected via indirect ELISA for the rRVGP and rabies vaccine. CEE-cELISAs were developed by using the monoclonal antibody clone 1–46-12, which recognizes a conformation epitope of the rabies G protein and rabies virus vaccine as antigens for ensuring the efficacy of immunization [11], while indirect ELISA was used to detect all epitopes to rRVGP and the whole rabies virus from coatings of the rRVGP and the rabies vaccine on ELISA plates, respectively. These data indicated that at least a single ID booster dose enhanced other epitopes to rRVGP and might enhance neutralizing activity, even in low-responder participants. This finding indicates that the number of vaccinations that an at-risk participant received was more important for avidity maturation than was the level of IgG and that an increase in avidity after boosting had a strong effect on the rRVGP. A booster vaccination seems to be necessary for optimal antibody maturation. Moreover, avidity maturation after priming and boosting is associated with the establishment of immunological memory [19], and because of this memory, protection against disease at that time is possible. Thus, further studies on B-cell memory maturation should be performed.

Our study has several limitations. Only VHVs and LVs that had to be vaccinated in the village were recruited; therefore, we enrolled these participants in each group in a cross-sectional study, and only some participants continued to participate. Therefore, some factors that should be considered are the presence of only male participants in the 2nd booster group and interindividual differences in the antibody response. Regardless of age, females tend to exhibit greater antibody responses than males, as well as higher basal immunoglobulin levels and greater B-cell numbers [20]. However, our study showed no difference in antibody levels or avidity indices between the 1st booster and 2nd booster groups. Another limitation is that the anti-rabies antibody levels measured by CEE-cELISA showed a very strong correlation with those in the RFFIT assay; however, the correlation values were detected in human sera only up to 100 EU/mL and showed no correlation with RFFIT as discussed in a previous study [11]. When the antibody concentration was greater than 100 IU/mL according to CEE-cELISA, the results could not be interpreted. However, the majority of the results were less than 50 IU/mL, and we measured the antibody levels against rRVGP in the same samples via indirect ELISA to confirm the response.

## Conclusion

Our study demonstrated that rabies antibodies persisted for more than 180 days and up to a year after cost-saving ID PrEP and the 1st or 2nd single ID booster dose, and an adequate antibody level was observed in more than 95% of participants by CEE-CELISA and 100% by indirect ELISA. However, the Thai National Government policy of using only one PrEP regimen for life-long protection of high-risk village volunteers should be reconsidered, given that less than approximately 50% of the people in this group did not have adequate antibodies after a year. Moreover, the avidity maturation of rabies-specific antibodies occurred during vaccination, especially after the 1st single ID booster dose. We demonstrated that a smaller ID booster dose was able to produce a sufficient immune response and enhance the maturation of anti-rabies antibodies. Our study suggested that one booster after PrEP would be effective at maintaining antibody levels before volunteers go to communities to vaccinate dogs. This finding is consistent with the recommendations of the WHO and Office International des Epizooties (OIE) for testing high-risk groups for rabies antibodies after two years and boosting if antibody levels are less than 0.5 IU/mL. Thus, this study presents a cost-effective rabies prevention strategy for high-risk individuals in resource-limited settings: a single-dose ID PrEP booster and a single-visit ID booster. This simplified schedule significantly reduces costs and eases implementation while potentially offering improved protection. However, ensuring equitable access for all at-risk individuals remains crucial. Existing schedule discrepancies highlight the need for bridging gaps and guaranteeing equal protection. This study underlines the importance of both cost-effective strategies and broader policy initiatives to address inequities and leave no one behind in the fight against rabies.

## Methods

### Study population

The study participants included village health volunteers (VHVs) and livestock volunteers (LVs) from the Mae Kha subdistrict, San Pa Tong district and Doi Lor subdistrict, Doi Lor Distinct, Chiang Mai Province, from February 2021 to September 2022. This study was approved by the ethics committee of the Faculty of Associated Medical Sciences, Chiang Mai University (number: AMSEC-63FB-005). All participants signed an informed consent before entering the study.

### Preexposure to rabies virus

The study population was divided into three groups: the PrEP, 1st booster and 2nd booster groups (Fig. 1). For participants entering the PrEP (naïve/never given rabies vaccination) stage, we collected blood samples from participants prior to 0.2 mL of the intradermal injection of the rabies vaccine (0.1 mL in each arm). After 7 days, the participants received another 0.2 mL of intradermal rabies vaccination. Blood samples were collected before day 0 (D0) and after vaccination on day 28 (D28) and day 180 (D180). For the 1st booster group, participants who had already received PrEP as a prime vaccination for one year received their 1st booster ID rabies vaccine at 0.1 mL. Blood samples were collected before the 1st booster vaccine, D0, and at D28 and D180 after the 1st booster immunization. For the 2nd booster group, participants who had received PrEP and the 1st booster ID vaccine received the 2nd booster shot of 0.1 mL of ID injection. Blood samples were also collected before the booster vaccine, at D0, D28 and D180 after the 2nd booster immunization. No signs of adverse reactions were reported after vaccination on day 28 in any of the groups.

### CEE-cELISA

CEE-cELISA was performed as previously described [11]. Briefly, 96-well plates (Thermo Fisher Scientific) were coated with SPEEDA^®^ rabies vaccine (Biovalys) overnight at 4 °C. The next day, the plates were washed with 0.05% Tween-20 in PBS 5 times prior to the addition of the blocking agent 3% BSA for 1 h at 37 °C. The blocking agent was removed before the addition of serially diluted serum samples at dilutions of 1:100, 1:200 and 1:400 together with diluted mouse mAb for competitive binding. The plates were incubated for 1 h at 37 °C, followed by washing and the addition of HRP-conjugated goat antimouse IgG as the secondary antibody. The plates were incubated at 37 °C for 1 h before removal and washing. To develop the samples, 3,3′,5,5′-tetramethylbenzidine (TMB) was added to the plates, followed by the addition of 1 N H_2_SO_4_ stop solution. The optimal density (OD) was measured at 450 nm using an Infinite F50 Plus (Tecan). The cutoff for adequate antibody levels was ≥ 0.7 EU/mL.

### Indirect ELISA and the avidity index

To determine the avidity index of the serum anti-rabies antibody, the protocol was adapted from Kewcharoenwong et al. [21]. The 96-well plates were coated overnight at 4 °C with the recombinant rabies virus glycoprotein rRVGP, which was kindly provided by Dr. Tadaki Suzuki [12]. The next day, the plates were washed three times with 0.1% Tween in PBS prior to the addition of 10% FBS in PBS as the blocking agent and incubated at room temperature for 2 h. The blocking agent was removed before the addition of 1:100 diluted serum (10% FBS in PBS with 0.05% Tween-20 was used as the diluent), after which the mixture was incubated at room temperature for 2 h. The plates were washed five times before treatment with 7 M urea solution in the designated wells for 30 min to remove any lightly bound IgG-antigen complex. The plates were washed again three times to remove the urea solution prior to the addition of 1:10,000 biotinylated mouse antihuman IgG (BD Biosciences) and a 1:1,000 streptavidin HRP (BD Biosciences) mixture diluted in the assay diluent as the secondary antibody for 1 h at room temperature. To develop the samples, TMB substrate was added for 10 min, followed by the addition of 1 N H_2_SO_4_ stop solution. The OD was measured at 450 nm using an Infinite F50 Plus (Tecan), and each sample value was interpolated with standard purified human IgG (1 µg/mL = 1000 AU/ml). The following formula was used for the calculation of avidity index percentages.

$$Affinity \; index \; (\%) = 100-\left[\frac{(IgG\; level \; untreated \; well-IgG\; level \; treated \; well)\times 100 }{IgG\; level \; untreated \; well}\right]$$

### Statistical analysis

The sets of results were compared between study groups using the Kruskal‒Wallis test with Dunn’s multiple comparisons test and the Chi-square test. The CIs were calculated using the exact binomial method (Clopper–Pearson method). All analyses were performed using GraphPad Prism 9 (GraphPad Software, San Diego, CA, USA). A p ≤ 0.05 was considered indicative of statistical significance. The power of each test was calculated by post hoc power analysis with a 95% confidence interval, and > 80% was acceptable for all experiments.

## Supplementary Information

Below is the link to the electronic supplementary material.

Supplementary file1 (PDF 423 KB)

## Data Availability

The datasets generated and analysed during the current study are available from the corresponding author upon reasonable request.

## Abbreviations

PrEP: Preexposure prophylaxis
CEE-cELISA: Cost-effective easy competitive enzyme-linked immunosorbent assay
rRVGP: Recombinant rabies virus glycoprotein
