# Supplementary material for: One-dose intradermal rabies booster enhances rabies antibody production and avidity maturation
Source: Med Microbiol Immunol. 2024 May 18;213(1):7. doi: 10.1007/s00430-024-00791-2 (PMC11102368; doi:10.1007/s00430-024-00791-2)
Supplement: Supplementary file 1 — Supplementary file1 (PDF 423 KB) [file 430_2024_791_MOESM1_ESM.pdf]

Additional data

One-dose intradermal rabies booster enhances adequate rabies antibody and the affinity maturation

Chidchamai Kewcharoenwong[1,2,3], Saranta Freeouf[1], Arnone Nithichanon[2,4], Wilaiwan Petsophonsakul[1,3], Sakorn Pornprasert[1], Woottichai Khamduang[1], Tadaki Suzuki[5], Taishi Onodera[6], Yoshimasa Takahashi[6], Ganjana Lertmemongkolchai[1,2]*

[1]Department of Medical Technology, Faculty of Associated Medical Sciences, Chiang Mai University, Chiang Mai, Thailand

[2]The Centre for Research & Development of Medical Diagnostic Laboratories, Faculty of Associated Medical Sciences, Khon Kaen University, Khon Kaen, Thailand

[3]Lanna Dog Welfare, Chiang Mai, Thailand

[4]Department of Microbiology, Faculty of Medicine, Khon Kaen University, Khon Kaen, Thailand

[5]Department of Pathology, National Institute of Infectious Diseases, Tokyo, Japan

[6]Research Center for Drug and Vaccine Development, National Institute of Infectious Diseases, Tokyo, Japan

*Correspondence:

Ganjana Lertmemongkolchai

ganjana.l@cmu.ac.th, ganja_le@kku.ac.th

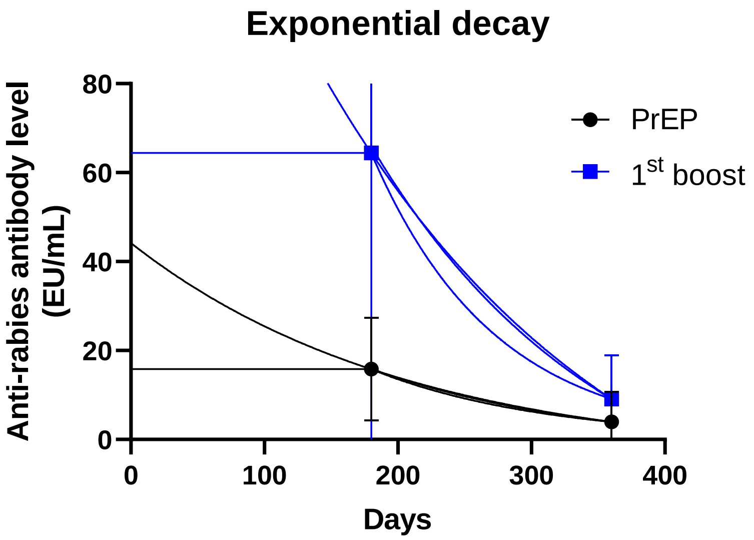

	PrEP	1st boost
Half Life	118.0	121.4

Additional Figure 1: Half-life of antibody. Statistical analysis was performed using one-phase exponential decay curves fitted to the decay phase of the selected response profiles (the lower limit of the 2-sided 95 % CI of the difference between groups was below the non-inferiority threshold).

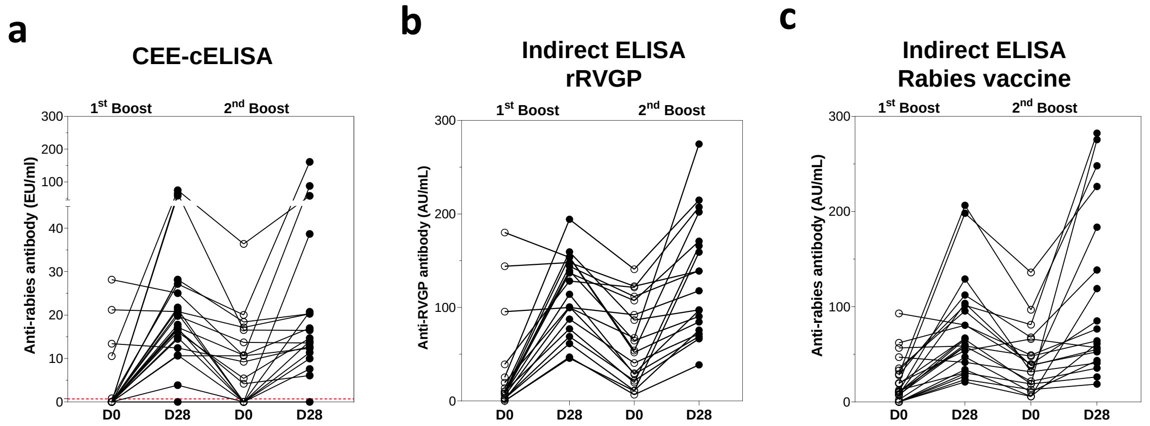

Additional Figure 2: Pattern of antibody response after 1st and 2nd boosters from 2nd boost group (n=19). (a) Rabies antibody level measured by CEE-cELISA. Horizontal dashed line indicates 0.7 EU/mL (indicator of adequate vaccination). (b) Antibody levels in response to recombinant rabies virus glycoprotein (RVGP) and (c) to rabies vaccine were measured by indirect ELISA. Each data point represents the median with interquartile range of each individual, with lines connecting datapoints from an individual.

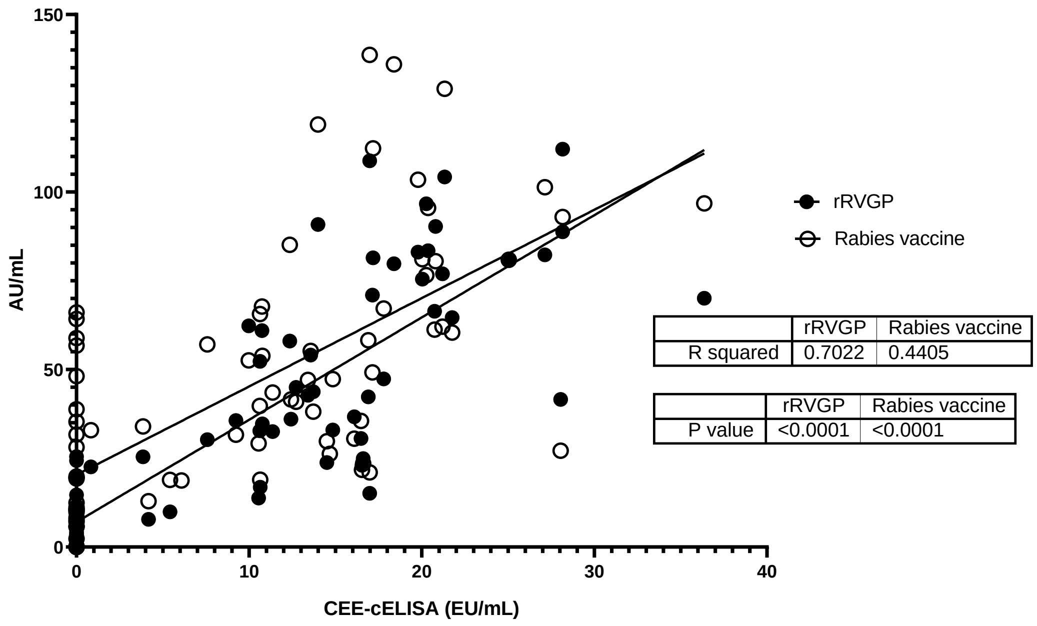

Additional Figure 3: Correlation between CEE-cELISA and indirect ELISA results. The same randomized samples (n=80) were tested by CEE-cELISA and indirect ELISA in response to recombinant rabies virus glycoprotein (rRVGP, black dot) and rabies vaccine (white dot). Each dot represents the value of each sample. Statistical analysis was performed using simple linear regression and the outliers was detected by ROUT method with Q set to 1% and excluded (n=5).
